# Cerebral Thrombophlebitis Complicating Coeliac Disease

**DOI:** 10.7759/cureus.66267

**Published:** 2024-08-06

**Authors:** Siham Zouiter, Dalal Bensabbahia, Meriem Atrassi, Abdelhak Abkari

**Affiliations:** 1 Pediatric Gastroenterology, Abderrahim El Harouchi Hospital, Ibn Rochd University Hospital, Casablanca, MAR; 2 Pediatric Gastroenterology, Abderrahim Harouchi Mother-Child Hospital, University Hospital Center Ibn Rochd, Casablanca, MAR

**Keywords:** thrombophilia, child, thrombosis, disease, celiac

## Abstract

Thromboembolic complications associated with coeliac disease are rare. They are dominated by abdominal venous thrombosis. However, cerebral thrombosis is exceptional. The research of the thrombotic risk factors is essential in coeliac disease. We report a clinical case illustrating cerebral thrombophlebitis due to antithrombin III deficiency with the presence of anticardiolipin antibodies complicating coeliac disease in a child.

## Introduction

Coeliac disease (CD) or gluten intolerance is an enteropathy caused by a hypersensitivity to gluten in genetically predisposed patients. The prevalence is estimated at 1/100 births. Thromboembolic manifestations are rare in coeliac disease [1-4]. The most frequent localization is abdominal venous thrombosis. Cerebral thrombosis is exceptional [1,2,5]. The pathogenesis of the association between coeliac disease and thrombosis is unclear. The aetiologies for high preponderance of venous thrombosis in coeliac disease are vitamin B12 and folate deficiency, genetic mutation in methylenetetrahydrofolate reductase, Protein S and C deficiency, and hyperhomocysteinemia [1,2,4]. Protein S and C deficiencies may be explained by the common vitamin K deficiency in coeliac disease [1,2,4,6]. CD being an autoimmune disorder is found to be associated with other immune disorders with high thrombogenic potential like antiphospholipid syndrome [4]. We present a clinical case illustrating this association between coeliac disease and cerebral thrombophlebitis.

## Case presentation

A 13-year-old girl was admitted to the gastroenterology and nutrition unit with severe malnutrition associated with uncontrollable vomiting and headaches. She had a history of chronic diarrhea and refractory iron deficiency anemia. The patient failed to respond to oral iron therapy during the preceding year. However, the anemia persisted. Clinical examination revealed a cachectic, apathetic child with pale skin. She weighed 16 kg (<-3 SD) for her height of 130 cm (<-3 SD), and her body mass index was 9.5 (<-3 SD), indicating severe malnutrition. Spindly limbs and flat buttocks were also observed on physical examination.

Laboratory investigations (Table 1) were ordered in view of her clinical presentation, revealing a microcytic hypochromic anemia with low ferritinemia at 8 ng/ml. Additionally, the hemogram objected a white blood cell count at 15000/mm^3^, and platelets at 196000/mm^3^. The prothrombin time was measured at 58%. Furthermore, the hydroelectrolytic imbalances showed hypokalemia at 2.6 mmol/l, hyponatremia at 125 mmol/l, hypophosphatemia at 0.60 mmol/l, and hypomagnesemia at 0.50 mmol/l. Significant hypoalbuminemia was observed at 16 g/l, accompanied by hypoproteinemia at 36 g/l. Infectious analysis was normal, including serologies [HIV, cytomegalovirus (CMV), Epstein-Barr Virus (EBV)], blood culture, stool test, three parasitological stool tests, and cytobacteriological urine exam.

**Table 1:** Laboratory results.

Laboratory tests	Reference values	Patient laboratory values
Hemoglobin (g/dl)	10.9-13.7	8
Mean corpuscular volume (%)	73-86	58
Mean corpuscular hemoglobin (pg)	27-32	16.8
White blood cell/mm^3^	7000-12000	15000
Neutrophil polynuclear/mm^3^	1500-7000	10000
Lymphocyte/mm^3^	1000-5000	3820
Platelets/mm^3^	150000-400000	196000
Ferritinemia (ng/ml)	15-200	8
Prothrombin time (%)	70-140	58
Hypokalemia (mmol/l)	3.5-5	2.6
Hyponatremia (mmol/l)	135-145	125
Hypophosphoremia (mmol/l)	0.74-1.52	0.60
Hypomagnesemia (mmol/l)	0.74-1.07	0.50
Hypoalbuminemia (g/l)	38-54	16
Hypoproteinemia (g/l)	64-83	36
Antitransglutaminase Ig A antibody (U/ml)	< 5	300
Antithrombin III (%)	80-120	24
Protein C (%)	70-140	72
Protein S (%)	70-130	62
Anti-cardiolipin antibodies Ig M (U/ml)	< 12	22
Anti-cardiolipin antibodies Ig G (U/ml)	< 10	0
Homocysteine (umo/l)	< 10	8
C3 complement (g/l)	0.90-1.80	0.95
C4 complement (g/l)	0.10-0.40	0.16
Antinuclear antibodies	< 80	< 80
Anti-DNA antibodies	< 10	0

**Figure 1:**
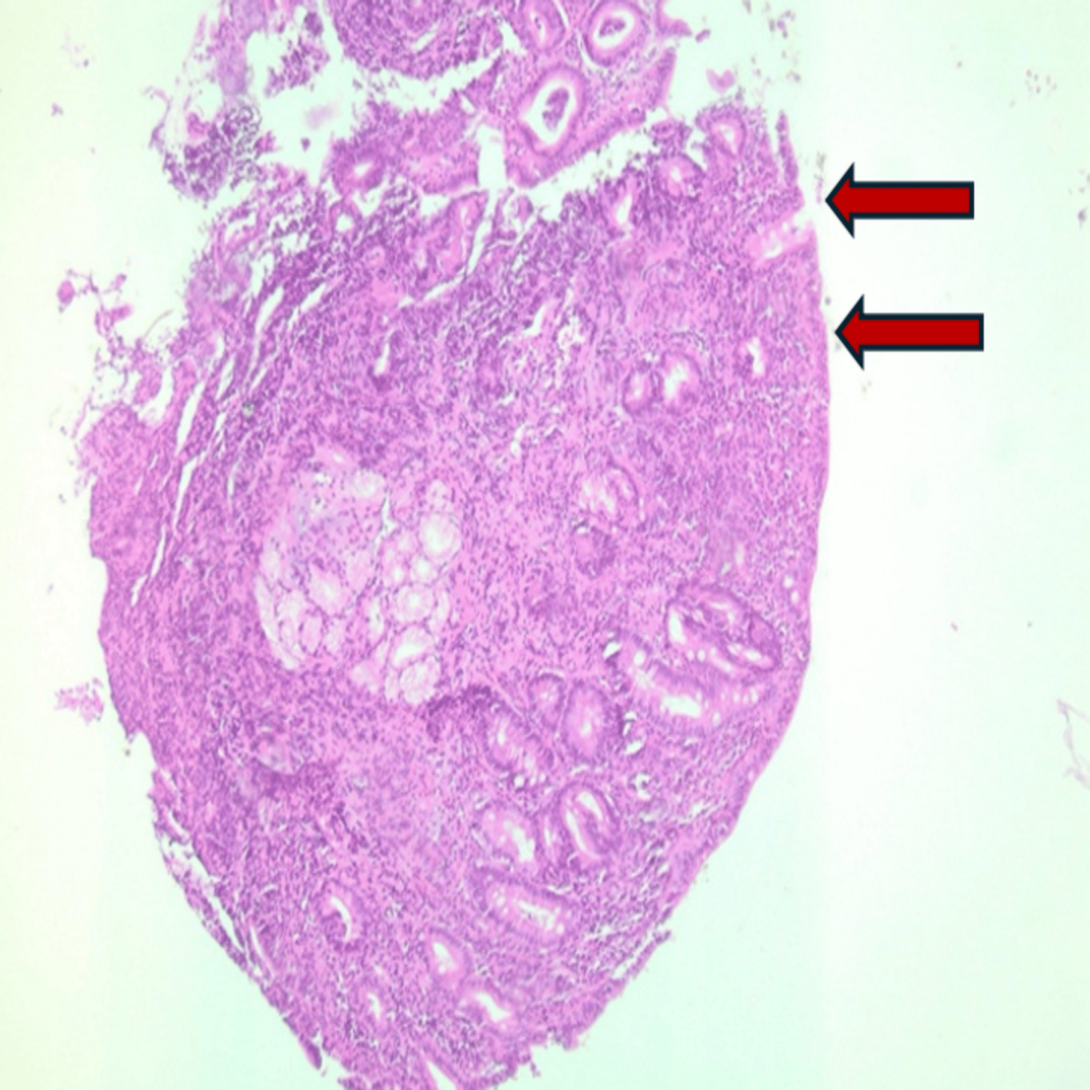
Total villous atrophy of the duodenal mucosa in our case report.

**Figure 2:**
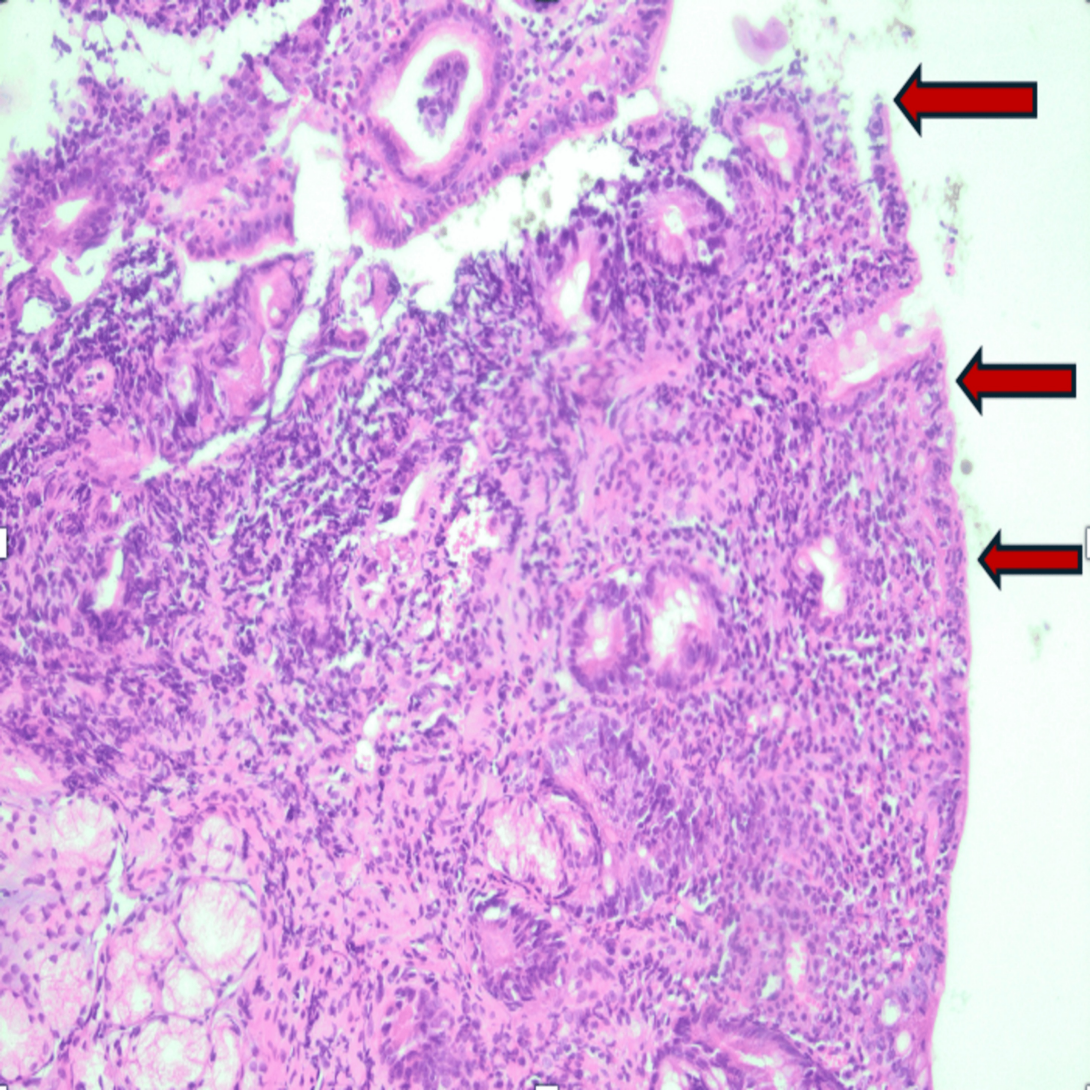
Increased number of intra-epithelial lymphocytes (40 lymphocytes/100 Enterocytes) in our case report.

Considering the clinical presentation, which includes chronic diarrhea and persistent iron deficiency anemia, an antitransglutaminase IgA antibody test was requested. They returned positive with a high level of 300 U/ml (Table 1). The gastrointestinal endoscopy was normal. the histological analysis revealed a total villous atrophy (Figure 1) and an increased number of intra-epithelial lymphocytes (Figure 2).

**Figure 3:**
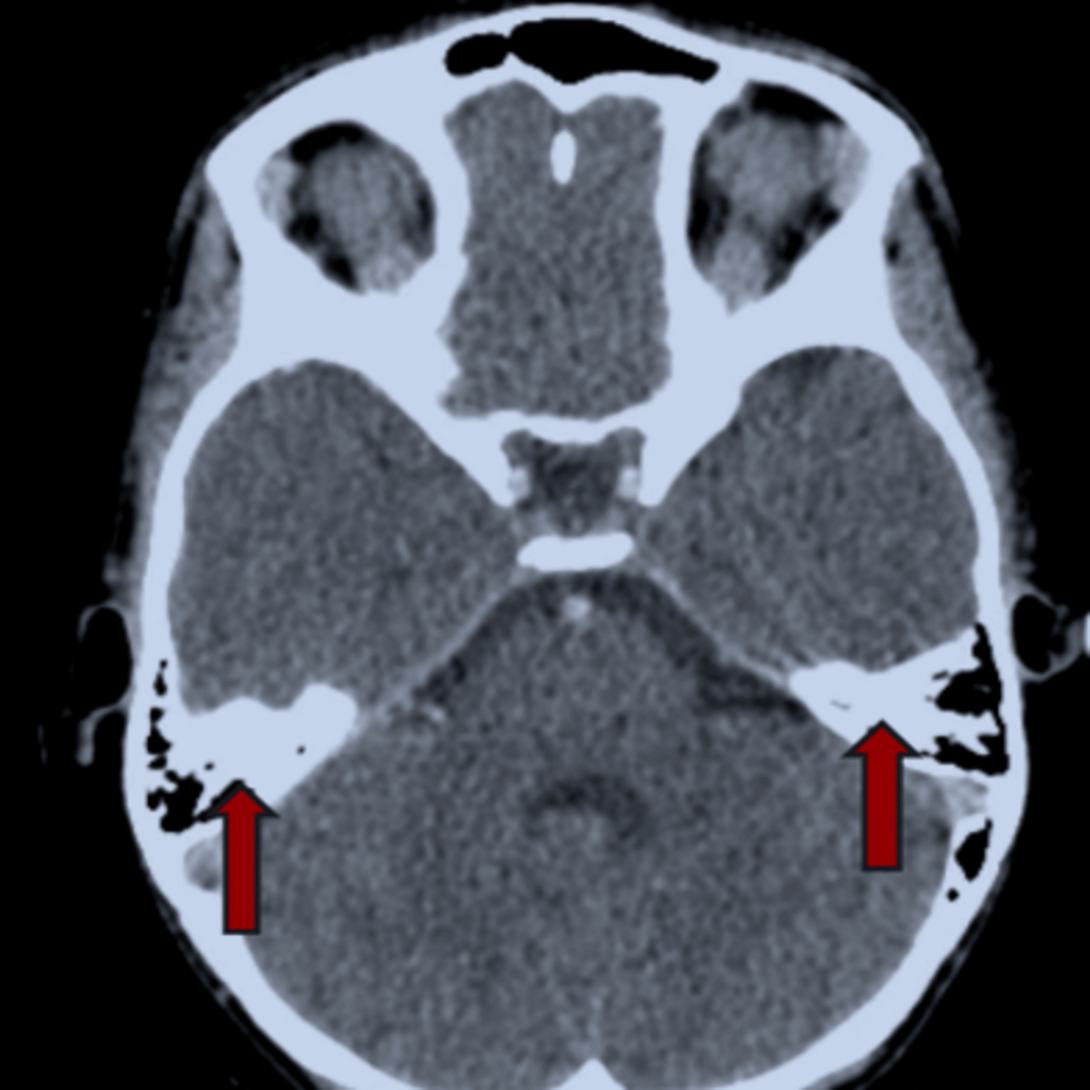
Cerebral scan showed thrombophlebitis of the cerebral venous sinus complicating celiac disease in our case report.

In the context of headaches accompanied by vomiting, a cerebral scan was performed. They revealed a thrombophlebitis in the right sigmoid sinus extending to the ipsilateral jugular bulb (Figure 3). The thrombophilia analysis was carried out, showing a reduced antithrombin III level at 24%, while protein C and S levels were normal. Immunological tests (C3, C4, homocysteine, antinuclear antibodies, and anti-DNA antibodies) returned normal. However, anticardiolipin Ig M antibodies returned positive.

The patient was promptly started on a gluten-free diet. Additionally, low molecular weight heparin (LMWH) was administered at 100 mg/kg/12 hours for cerebral venous thrombosis. The short outcomes were marked by thrombocytopenia. Consequently, Fondaparinux, one of a new class of injectable anticoagulants that selectively inhibit a specific coagulation enzyme, factor Xa, was started at a dosage of 2.5 mg/12 hours for six weeks. The nutritional rehabilitation was started in parallel. The initial nutritional intake was 25 kcal/kg/day (400 kcal/day), then gradually increased to 2000 kcal/day, accompanied by zinc, vitamin A, vitamin K, thiamine, and magnesium supplementation, and oral iron supplementation. A spectacular evolution was observed on the gluten-free diet, with the disappearance of diarrhea and a weight gain of 3 kg (20%) after one week. We noted a complete repermeabilization of the right transverse sinus and resolution of internal jugular vein thrombophlebitis within four weeks. In addition, platelet levels returned to normal on fondaparinux, enabling the transition to oral vitamin K antagonists (VKA) with a favorable outcome.

## Discussion

Coeliac disease is an autoimmune enteropathy associated with gluten intolerance in genetically predisposed patients. Clinical manifestations are highly varied. Complications of coeliac disease frequently include severe malnutrition, growth retardation, vitamin, and iron deficiencies [1,2]. Thrombophlebitis during coeliac disease has been reported in the literature, especially in adults [3]. In contrast, it is a rare condition in children [1,3]. Most often, abdominal venous thrombosis involving the suprahepatic veins, the spleno-mesenteric venous trunk, and the central nervous system are reported [3,6,7]. Occasionally, arterial thrombosis, notably myocardial infarction, and mesenteric artery infarction, has been reported [3].

The pathogenesis of the association between coeliac disease and thrombosis is unclear [6]. Thrombophilia risk factors associated with thrombosis in coeliac disease include protein S, C, and antithrombin III deficiency [1,3,7]. Protein S and C deficiencies may be explained by the common vitamin K deficiency in coeliac disease. Vitamin K, as a coenzyme in glutamic acid carboxylation, is a cofactor for the synthesis of protein C and its cofactor protein S. Deficiency of protein C and protein S leads to uncontrolled coagulation activation and consequently thrombosis [7,8]. Pantic et al. [7] revealed Protein C and S deficiency in 31.6% of patients. Antithrombin is a protein inhibiting thrombin and factors (Xa, IXa, XIa). Consequently, antithrombin III deficiency predisposes to venous thrombosis [1,3,9].

Homocysteine is an amino acid resulting from the catabolism of methionine. The enzyme methylenetetrahydrofolate reductase and vitamin B destroys homocysteine. Consequently, the deficiency in vitamins B6, B9 or B12 alters methionine synthesis and leads to hyperhomocysteinemia. Furthermore, it is connected to different procoagulant states, such as platelet activation, endothelial dysfunction, oxidative stress, and reduced levels of protein C and antithrombin [7]. Hyperhomocysteinemia is noted in coeliac disease, with a prevalence of 20% [9].

Antiphospholipid syndrome is an autoimmune disease combining thrombosis and antiphospholipid antiantibodies (anti-beta-2-glycoprotein antibodies and/or anti-cardiolipin antibodies). A prospective study by Karoui et al. [10] compared the prevalence of antiphospholipid antibodies in 50 patients with coeliac disease versus 50 controls. There was no difference between the two groups in terms of the prevalence of anti-beta-2-glycoprotein antibodies and anticardiolipin antibodies (Ig A and Ig M). Only IgA isotype anticardiolipin antibodies were more frequently detected in the coeliac disease group but were not responsible for any thrombosis [10]. The exact mechanism by which Ig A isotype anticardiolipin antibodies are produced is unclear. In coeliac disease, increased apoptosis of enterocytes was involved in epithelial cell loss and mucosal atrophy. Thus, exposure of cardiolipin on enterocyte apoptotic blebs could trigger beta-2-glycoprotein I (β2GPI) fixation and then production of antiphospholipid of Ig A isotype, because the immune response occurs in the microenvironment of the intestine [10].

In the Berthoux et al. study [3], one patient had very significant thrombocytosis (>1000000/mm^3^), which may have favored thrombosis of the superficial femoral vein. Thrombocytosis is common in coeliac disease (60%) and reflects several factors, including iron deficiency and inflammation [11]. In contrast, the platelet count was normal (196000/mm^3^) in our case report.

The gluten-free diet led to protein C, protein S and homocysteine level normalization. This could be because of the reduction in intestinal inflammation, and the recovery of the mucosal layer. These phenomena restore the reabsorption of nutrients (B12, B9, and B6), normalization of homocysteine and iron levels [7]. The anticoagulation therapy is recommended in all coeliac disease patients with thrombosis unless there are strict contraindications [7]. Pantic et al. [7] administered an anticoagulation therapy to all patients with a good outcome (100% survival). However, the duration of therapy remains unclear and has to be answered in more prospective studies in the future [12,13]. This case report is a rare medical observation of cerebral thrombophlebitis with antithrombin III deficiency and the presence of anticardiolipin Ig M antibodies complicating coeliac disease in children.

## Conclusions

Cerebral thrombosis in coeliac disease is rare. The mechanism of this association remains unclear. It prompts investigation of certain associated risk factors, notably hyperhomocysteinemia, protein S and C, antithrombin III deficiencies, and antiphospholipid syndrome. Coeliac disease and antiphospholipid syndrome are autoimmune diseases. Anticardiolipin Ig A antibodies were more frequently detected in coeliac disease patients but were not responsible for any thrombosis. Consequently, we can consider antiphospholipid syndrome a comorbidity of coeliac disease. Early treatment with anticoagulants, combined with a gluten-free diet and correction of deficiencies, leads to a favorable outcome. Prospective studies are needed to understand the pathogenesis of this association, the duration of the anticoagulant treatment, and the outcomes of cerebral thrombosis in coeliac disease.
